# Prediction of Cerebral Aneurysm Hemodynamics With Porous-Medium Models of Flow-Diverting Stents via Deep Learning

**DOI:** 10.3389/fphys.2021.733444

**Published:** 2021-09-17

**Authors:** Gaoyang Li, Xiaorui Song, Haoran Wang, Siwei Liu, Jiayuan Ji, Yuting Guo, Aike Qiao, Youjun Liu, Xuezheng Wang

**Affiliations:** ^1^Institute of Fluid Science, Tohoku University, Sendai, Japan; ^2^Department of Radiology, Shandong First Medical University and Shandong Academy of Medical Sciences, Tai’an, China; ^3^Faculty of Environment and Life, Beijing University of Technology, Beijing, China

**Keywords:** cerebral aneurysm, hemodynamics, flow-diverting stent, porous-medium, deep learning

## Abstract

The interventional treatment of cerebral aneurysm requires hemodynamics to provide proper guidance. Computational fluid dynamics (CFD) is gradually used in calculating cerebral aneurysm hemodynamics before and after flow-diverting (FD) stent placement. However, the complex operation (such as the construction and placement simulation of fully resolved or porous-medium FD stent) and high computational cost of CFD hinder its application. To solve these problems, we applied aneurysm hemodynamics point cloud data sets and a deep learning network with double input and sampling channels. The flexible point cloud format can represent the geometry and flow distribution of different aneurysms before and after FD stent (represented by porous medium layer) placement with high resolution. The proposed network can directly analyze the relationship between aneurysm geometry and internal hemodynamics, to further realize the flow field prediction and avoid the complex operation of CFD. Statistical analysis shows that the prediction results of hemodynamics by our deep learning method are consistent with the CFD method (error function <13%), but the calculation time is significantly reduced 1,800 times. This study develops a novel deep learning method that can accurately predict the hemodynamics of different cerebral aneurysms before and after FD stent placement with low computational cost and simple operation processes.

## Introduction

Currently, strokes, including cerebral aneurysms, have the highest mortality rate of diseases worldwide (Tsai et al., 2018). Flow-diverting (FD) stent placement is a common and effective method for treating cerebral aneurysms (Li Y. et al., 2018). A large number of studies have shown that analyzing hemodynamics (such as flow velocity field, pressure field, etc.) in cerebral aneurysms before and after FD stent placement has a positive guiding significance for selecting and optimizing treatment options (Sforza et al., 2009; Munarriz et al., 2016; Patzig et al., 2017; Panchendrabose et al., 2021).

Computational fluid dynamics (CFD) is gradually used in the calculation of hemodynamics. Based on the given boundary conditions and geometric information of the model, CFD can solve the conservation equations of mass, momentum and energy on the discrete meshes by the Navier–Stokes equation, and then obtain the numerical solutions of the flow field hemodynamics. However, when calculating the hemodynamics of cerebral aneurysm with FD stent, CFD often needs complex operation processes and high computational costs. This is due to the construction and placement simulation of a fully resolved FD stent, which usually requires professional operation skills and long-time iterative calculation (Zhang et al., 2017). As an alternative of fully resolved FD stent, porous modeling of FD structure has been introduced and applied to a certain extent (Li et al., 2017; Dazeo et al., 2020). The porous-medium model represents FD’s tubular mesh as a thin layer with certain permeability. Owing to the drastic decrease of numerical meshes, computational time can decrease. However, requirement of high professional skills still remains. Therefore, it is necessary to develop a method with simple operations and low computational costs.

Machine learning or deep learning technique has been used for fluid dynamics field such as modeling of turbulent flow or further direct estimation of flow field. With the support of high-performance GPU computing clusters and network structures, deep learning can extract the underlying features through neural network to build abstract high-level features or attribute features, and then achieve faster and more accurate pattern classifications or regression tasks than traditional methods (Li G. et al., 2018; Li et al., 2019, 2021; VanRullen and Reddy, 2019; Kiesow et al., 2021). Guo et al. (2016) used a deconvolution network to predict the change of 2D flow field around obstacles with simple geometry. Gamahara and Hattori (2017) established a functional relation between the grid-scale flow field and the subgrid-scale stress using an artificial neural network. Liang et al. (2020) developed a deep learning method for predicting the internal hemodynamics of 3D idealized thoracic aortic models. Most of deep learning methods proposed in previous studies have limited data dimensions and resolutions such as voxel-wise or pixel-wise, and this makes it difficult to characterize the complex geometric structure and internal flow field. The cerebral aneurysms of different patients have great morphological changes (Skodvin et al., 2017). In addition, before and after FD stent placement, there are complex 3D flow fields like a vortex in the cerebral aneurysm models (Meuschke et al., 2018). In order to apply deep learning to realize the cerebral aneurysm hemodynamic prediction, a flexible flow field representation data format needs to be selected to accurately characterize the different geometric shapes and the complex flow field distributions. At the same time, a deep learning network structure that can handle this new data format must also be proposed.

In this study, we evolved the hemodynamics prediction network for the cerebral aneurysms flow including FD stent layers. We established the aneurysm hemodynamics point cloud data sets based on CFD simulation of ideal side-wall aneurysms with or without porous-medium models. Corresponding to the data set, a deep learning network with double input and sampling channels was proposed. The velocity and pressure prediction errors were calculated to evaluate deep learning performance. Compared with the previous deep learning method, our deep learning method can flexibly characterize the complex geometry including fluid domain and FD domain in the same scheme and realize the flow field prediction without identifying the point cloud belonging to FD domain. Compared with the traditional CFD approach to calculate the aneurysm hemodynamics with FD stent, our deep learning method could avoid the stent construction and placement simulation, which greatly simplified the operation process and reduces the computational cost.

## Materials and Methods

### Creation of Date Sets

The data sets used in this study were from the CFD simulation hemodynamics of the side-wall aneurysms before and after FD stent placement. Therefore, the creation of data sets included three steps: the generation of cerebral aneurysm models, the flow field calculation of cerebral aneurysm model before and after FD stent placement, the point clouds extraction and data sets establishment.

#### Generation of Cerebral Aneurysm Models

**FIGURE 1:**
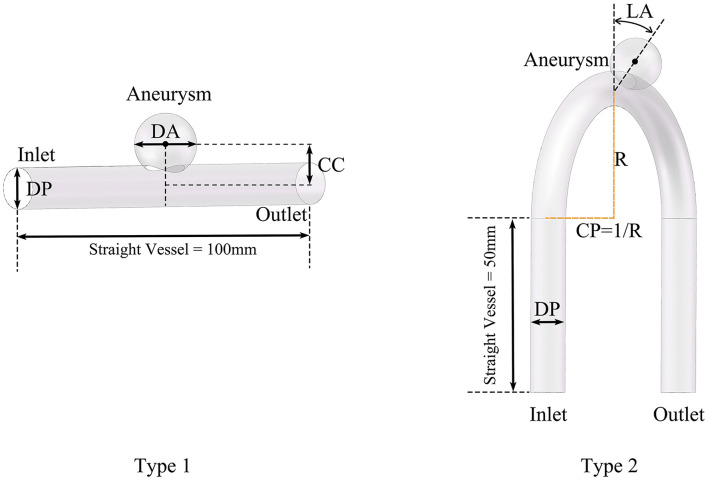
Definition of morphological parameters and generation of cerebral aneurysm models.

We divided the models into two types according whether the parent artery was straight or curved, as shown in Figure 1. Based on previous studies on the morphology of cerebral aneurysms (Chung et al., 2018; Detmer et al., 2018; Zhang et al., 2018; Amigo and Valencia, 2019) we selected five morphological parameters and randomly selected values within a reasonable range to generate 500 aneurysm models (100 Type 1 models and 400 Type 2 models), as shown in Figure 1 and Table 1. The five morphological parameters were the diameter of aneurysm (DA), distance from aneurysm center point to parent artery center line (CC), diameter of parent artery (DP), curvature of parent artery (CP), and location of aneurysm (LA).

**TABLE 1:** The range of morphological parameters.

**Parameter**	**DA (mm)**	**CC (mm)**	**DP (mm)**	**CP (mm** ^–^ **^1^)**	**LA**
Range	8.0–12.0	6.0–8.0	8.0–12.0	0.01–0.033	0–180°

#### Flow Field Calculation of Cerebral Aneurysm Model Before and After FD Stent Placement

The flow field hemodynamic inside the aneurysm model was calculated by the CFD method. Under the assumptions of incompressible Newtonian laminar flow, constant density and no slip wall, the continuity equation and Navier–Stokes equation were solved to calculate the flow field velocity and pressure:


(1) $$\frac{\partial V_{x}}{\partial x}+\frac{\partial V_{y}}{\partial y}+\frac{\partial V_{z}}{\partial z}=0$$

(2) $$\rho \,\left(\frac{\partial V_{x}}{\partial t}+V_{x}\,\frac{\partial V_{x}}{\partial x}+V_{y}\,\frac{\partial V_{x}}{\partial y}+V_{z}\,\frac{\partial V_{x}}{\partial z}\right)=\mu \,\left[\frac{\partial^{2}V_{x}}{\partial x^{2}}+\frac{\partial^{2}V_{x}}{\partial y^{2}}+\frac{\partial^{2}V_{x}}{\partial z^{2}}\right]-\frac{\partial P}{\partial x}$$

(3) $$\rho \,\left(\frac{\partial V_{y}}{\partial t}+V_{x}\,\frac{\partial V_{y}}{\partial x}+V_{y}\,\frac{\partial V_{y}}{\partial y}+V_{z}\,\frac{\partial V_{y}}{\partial z}\right)=\mu \,\left[\frac{\partial^{2}V_{y}}{\partial x^{2}}+\frac{\partial^{2}V_{y}}{\partial y^{2}}+\frac{\partial^{2}V_{y}}{\partial z^{2}}\right]-\frac{\partial P}{\partial y}$$

(4) $$\rho \,\left(\frac{\partial V_{z}}{\partial t}+V_{x}\,\frac{\partial V_{z}}{\partial x}+V_{y}\,\frac{\partial V_{z}}{\partial y}+V_{z}\,\frac{\partial V_{z}}{\partial z}\right)=\mu \,\left[\frac{\partial^{2}V_{z}}{\partial x^{2}}+\frac{\partial^{2}V_{z}}{\partial y^{2}}+\frac{\partial^{2}V_{z}}{\partial z^{2}}\right]-\frac{\partial P}{\partial z}$$

Where *V*_*x*_, *V*_*y*_, and *V*_*z*_ were the velocity components in the flow field in x, y, and z directions, respectively. P was the flow field pressure, ρ was the fluid density, and μ was the fluid viscosity. Regarding the material properties of vessels and blood, ρ was 1,050 kg/m^3^ and μ was 0.0035 Pa⋅s.

In this study, the simulation was set as a steady simulation, which meant that boundary conditions were constants independent of time. The purpose of this study was to use deep learning to analyze and reproduce the relationship between model geometry and hemodynamics. Therefore, we set the boundary conditions within a reasonable range. The boundary conditions of all models were uniformly set as 0.004375 kg/s at the inlet and zero pressure at the outlet.

As mentioned above, we used porous media method to represent FD stent. The shape of the porous media layer was based on the shape of the surface of the blood vessel wall at the intersection plane between the sphere and the parent artery. The thickness of the porous media layer was 150 μm. The resistance S of the porous media layer could be expressed as:


(5) $$S_{x}=-\left(\frac{\mu }{K}\,V_{x}+C\,\frac{1}{2}\,\rho \,\left|V\right|\,V_{x}\right)$$

(6) $$S_{y}=-\left(\frac{\mu }{K}\,V_{y}+C\,\frac{1}{2}\,\rho \,\left|V\right|\,V_{y}\right)$$

(7) $$S_{z}=-\left(\frac{\mu }{K}\,V_{z}+C\,\frac{1}{2}\,\rho \,\left|V\right|\,V_{z}\right)$$

Where the specific values of permeability K and loss coefficient C were 0.001489 mm^2^ and 7,665 m^–1^, which was obtained according to FD stent properties (Li Y. et al., 2018).

All models were established using Solidworks (France) commercial software. After the 3D models were pre-processed, ANSYS-Meshing (United States) commercial software was used for making mesh to generate the computational models. In the final step, we used ANSYS-CFX for simulation and got the velocity and pressure distribution results.

#### Point Clouds Extraction and Data Set Establishment

Using ANSYS or other simulation software, the hemodynamic results from CFD could be directly transformed into a point cloud format. The point cloud was extracted from the connection points (usually called nodes) of the CFD meshes, so it inherited the CFD meshes to resolve the geometric structure of the model. The spatial distribution of the point cloud varied with the geometry of different models. Compared with the fixed format samples that were used in the previous deep learning methods (Guo et al., 2016; Liang et al., 2020), the density and number of point clouds changed according to the geometric structure of the model, which meant that there were stronger characterization capabilities. Under reasonable settings, the point cloud could represent the geometry and hemodynamics of the model, which was proved by the mesh independence test result below.

Two kinds of point clouds were extracted—namely, the model point cloud{*M*_*i*_|*i* = 1,2,3…,*N*_1_} and query point cloud{*Q*_*i*_|*i* = 1,2,3…,*N*_2_}. *N*_*1*_ and *N*_*2*_ were the total number of model points and query points in one model, respectively. The model point cloud was extracted from the outermost mesh nodes, which contained only the overall geometric information (the set of coordinates in x, y, and z directions of each point) of the cerebral aneurysm. The query point cloud was extracted from the mesh nodes inside the model. In addition to the spatial distribution information, the query point cloud also contained the velocity and pressure value corresponding to each point.

We extracted two kinds of point clouds of all models before and after FD stent placement and then constructed four aneurysm hemodynamic data sets: preoperative, postoperative, velocity, and pressure fields data sets. We randomly divided each data set into a training set (a total of 450, including 90 type 1 and 360 type 2 models) and a test set (a total of 50, including 10 type 1 and 40 type 2 models) according to the proportion of 9:1. During the pre-experiment, we calculated the prediction error of multiple randomly divided training/test set combinations (the proportion is 9:1) on the network. There was no significant difference in the errors of different data sets, which could prove the robustness of the scheme. The four data sets needed to be used separately as inputs to train the network. Velocity or pressure predictions also needed to be carried out separately using the corresponding trained network.

### The Proposed Deep Learning Network

**FIGURE 2:**
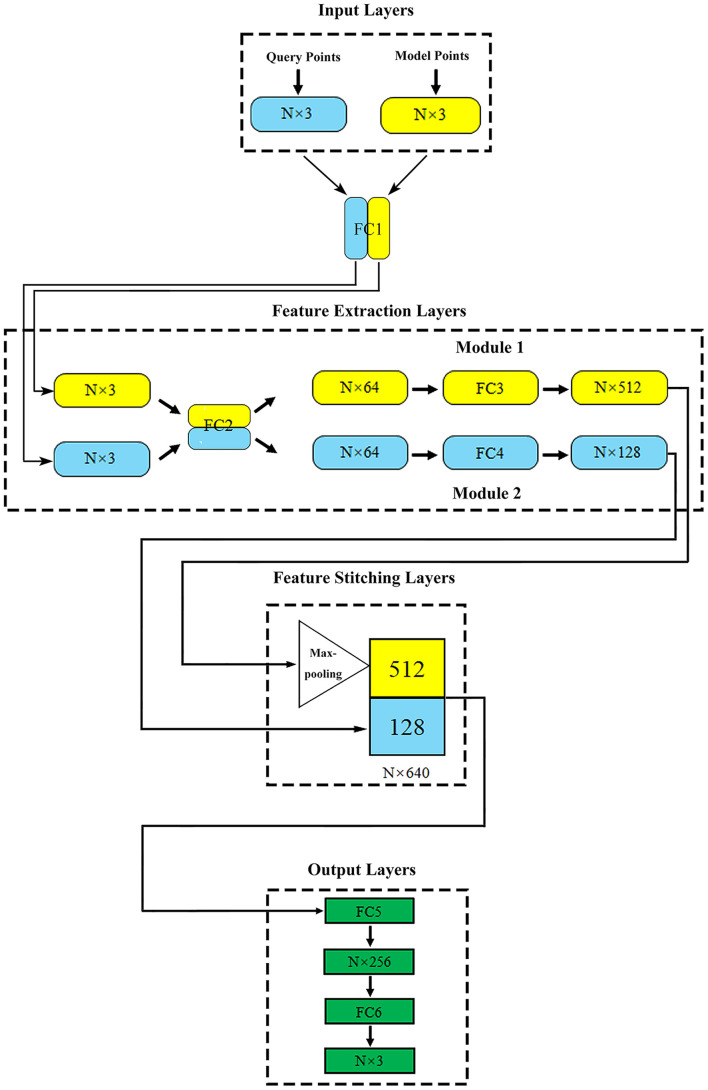
The proposed network structure. N (in millions) represents the total number of points contained in the query point cloud and the model point cloud in one model.

Based on the properties of created data sets, we adopted an optimized network structure for flow prediction on flexible structure of point cloud (Li et al., 2021) which was developed based on PointNet (Qi et al., 2017). Its specific details and parameter settings were shown in Figure 2.

The training process and operation principle of our network could be described as follows:


(8) $$f_{1}\left(Q_{H\,1},Q_{H\,2},\ldots ,Q_{H\,N_{2}}\right)=\gamma^{\circ }[\left(h_{1}\left(Q_{1}\right),\ldots ,h_{1}\left(Q_{N_{2}}\right)\right)+G\left(h_{2}\left(M_{1}\right),\ldots ,h_{2}\left(M_{N_{1}}\right)\right)]$$

Where *f*_1_(*Q*_*H*1_,*Q*_*H*2_,…,*Q*_*HN*_2__) represented the predicted output of hemodynamics corresponding to each point of the input query point cloud. In the training process, query point cloud {*Q*_*i*_|*i* = 1,2,3…,*N*_2_} (x, y, z coordinates and corresponding velocity or pressure value) and model point cloud {*M*_*i*_|*i* = 1,2,3…,*N*_1_} (x, y, z coordinates) were used as network input. *h*_*1*_ and *h*_*2*_ represented the feature extraction methods represented by module 2 and module 1, respectively. *h*_*1*_ extracted the local coordinates and corresponding hemodynamic features of each point in the input query point cloud, which were used as teaching signals in the training process. *h*_*2*_ extracted the overall geometric features represented by the input model point cloud. G was the symmetric function (MaxPooling). The addition of *h*_*1*_ and *h*_*2*_ represented the feature stitching operation, that was, the two features were stitched together to realize the further prediction function of the output layer. γ represented high-dimensional abstract feature extraction. Furthermore:

The proposed deep learning network consisted of four parts: input layers, feature extraction layers, feature stitching layers, and output layers.

Input layers: this part included two input layers, which were used to import the model point cloud and query point cloud of aneurysm, respectively.

Feature extraction layers: two feature extraction modules were directly connected with two input layers. Module 1 extracted the geometric features of aneurysm as global features. Module 2 extracted the spatial coordinates of the internal query point cloud and the corresponding flow velocity or pressure field as local features. In order to enhance the relationship between global features and local features, fully connected layer 1 (FC1) and fully connected layer 2 (FC2) shared the weights, which meant that they shared the same extraction method in the primary extraction stage. The symmetric function Maxpooling layer after module 1 could solve the disorder of input point cloud (Qi et al., 2017; Ghahremani et al., 2020). After feature extraction, the global and local features of aneurysm were abstracted as N vectors of 512 dimensions (N × 512) and 128 dimensions (N × 128), respectively.

Feature stitching layers: In this part, vectors representing global and local features could be stitched together to form an N × 640 (512+128) dimensional feature. With the global feature as the constraint and the local feature as the teachers’ signal, the spatial relationship was effectively introduced to help the network attain correspondence between the model geometry and the flow velocity or pressure distribution point by point.

Output layers: this part outputted the flow velocity field corresponding to the internal query point cloud.

The difference between our deep learning method and the original PointNet was that there were two types of input point clouds and two corresponding input layers of the network. The original PointNet had only one type of input point cloud and a single input layer. Our design could make the network extract the geometric structure and flow field features of the training data better, which was proved by an ablation experiment (comparing the prediction error between our network and the original PointNet) and a control experiment (with or without “shared weight”) in our previous study (Li et al., 2021).

For other details, we used the mean absolute error as the loss function. Since the number of points in a single model reached the level of 0.3 million, the parameter batch size was set to 1, which meant that the input for one training iteration was all model points and query points in a single model. The optimizer was Adam with learning rate = 0.001, ε = 0.001, ρ1 = 0.9, ρ2 = 0.999, and δ = 1E–8 (Kingma and Ba, 2014). The training environment was Tensor Flow (v2.0.0rc), Python (v3.6) on a Nvidia GeForce GTX 1080 Ti GPU. As mentioned above, four data sets needed to be separately trained as inputs for the network. During the training process, we saved the optimal network parameter configurations for each training set. As for the test process, it was necessary to take the model point cloud (x, y, z coordinates) and query point cloud (only x, y, z coordinates) in the test sets as the input. The trained network could achieve the rapid output of the corresponding velocity or pressure value of the query point cloud. The further explanation of the usage details of data sets during network training and testing is shown in Table 2.

**TABLE 2:** The usage details of data sets during network training and testing.

**Network**	**Data set**	**Input**	**Output**	**Number of models**	**Number of points (variables) in a model**
Training	Training set	Model point cloud (x, y, z coordinates) + query point cloud (x, y, z coordinates and hemodynamic values calculated by CFD)	Deep learning hemodynamic prediction values corresponding to input query point cloud	450	About 0.1 (Type 1 model) –0.3 (Type 2 model) million
Testing	Test set	Model point cloud (x, y, z coordinates) + query point cloud (only x, y, z coordinates)	Deep learning hemodynamic prediction values corresponding to input query point cloud	50	About 0.1 (Type 1 model) –0.3 (Type 2 model) million

### Definition of Error Functions

In order to evaluate the performance of our deep learning method, referring to the previous research (Liang et al., 2020; Li et al., 2021), we defined two error functions (ERR), normalized mean absolute error (NMAE), and mean relative error (MRE), to quantitatively compare the difference between deep learning and CFD flow field calculation results. The ERR were defined as:


(9) $$N\,M\,A\,E=\frac{1}{N_{2}}\,\frac{\sum_{i=1}^{N_{2}}\left|Q_{i}-{\hat{Q}}_{i}\right|}{M\,a\,x\,\left|Q\right|-M\,i\,n\,\left|Q\right|}\times 100\%$$

(10) $$M\,R\,E=\frac{1}{N_{2}}\,\sum_{i=1}^{N_{2}}\frac{\sqrt{{\left(Q-{\hat{Q}}_{i}\right)}^{2}}}{\sqrt{Q_{i}^{2}}}\times 100\%$$

Where *Q*_*i*_ and ${\hat{Q}}_{i}$ represented the velocity or pressure value of the ith query point calculated by CFD and deep learning method, respectively. Max|*Q*| and Min|*Q*| represented the maximum and minimum magnitude of velocity or pressure in one model, respectively. NMAE reflected the performance of deep learning method in the whole model and MRE focused on evaluating the prediction accuracy by testing the error of each point.

## Results

**FIGURE 3:**
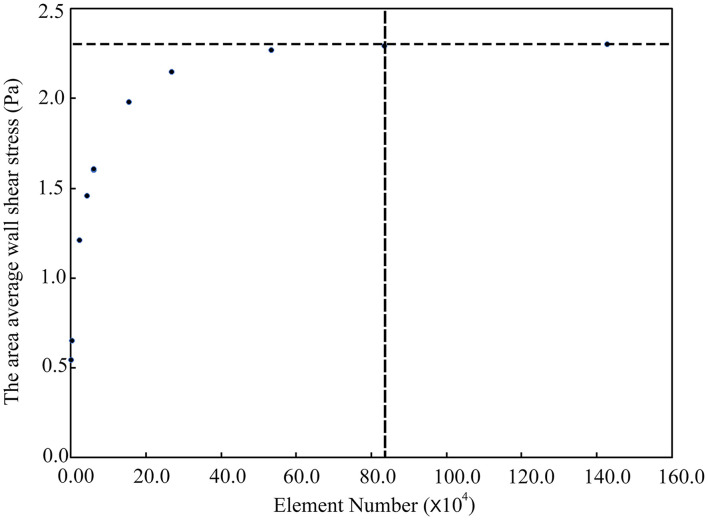
The mesh independence test.

Due to the relatively simple geometry and internal flow field of the type 1 models, we took the type 2 models as examples to show the mesh independence test result, as shown in Figure 3. When the number of meshes exceeded 0.83 million, the simulation results could be considered stable. In this study, the number of meshes in different aneurysm models (type 2) was about 1.6 million. So the point cloud extracted from the original CFD meshes could accurately represent the spatial geometry of the aneurysm models.

**FIGURE 4:**
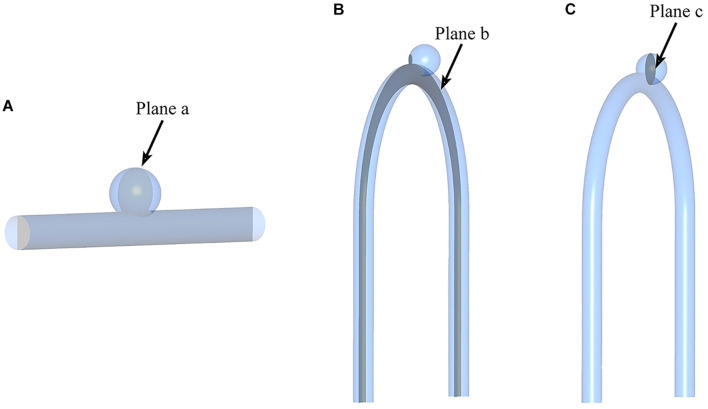
Sketch of measurement planes. For Type 2 models, use a sample with an LA of 30°. The results shown in Figures 5, 6 are the velocity and pressure distribution on the section shown in positions **(A–C)**. **(A)** Type 1 model symmetry plane; **(B)** Type 2 model parent artery symmetry plane; **(C)** Type 2 model aneurysm cross section.

To visually show the prediction results of deep learning, we randomly selected the models in the test sets and set the cross section, as shown in Figure 4.

**FIGURE 5:**
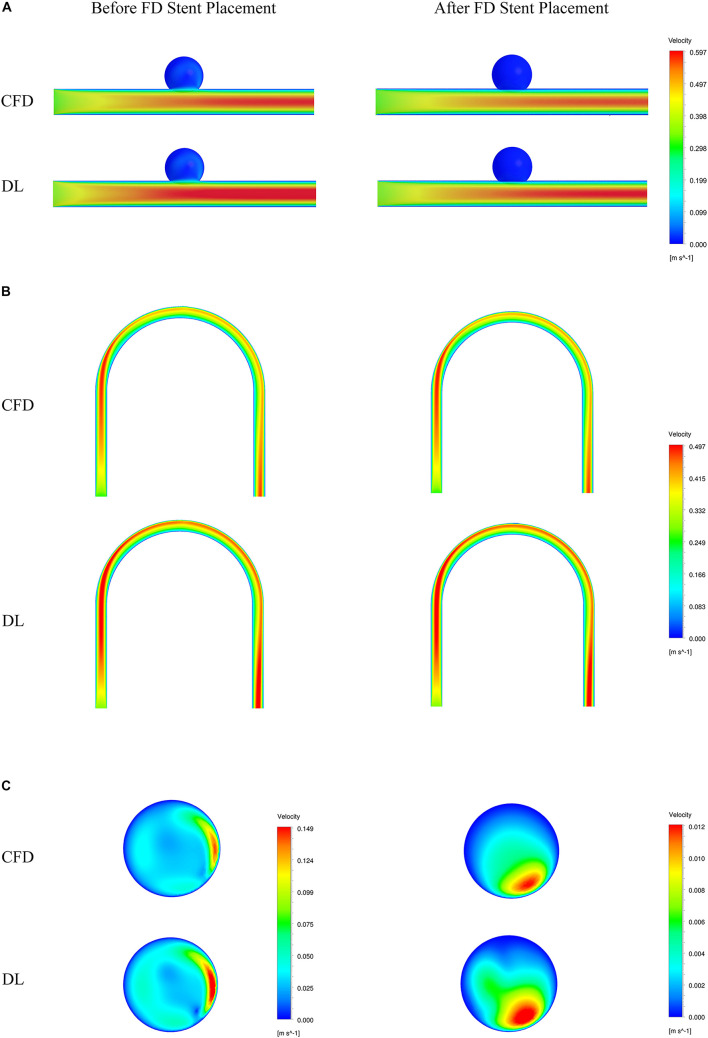
Comparison of velocity fields from CFD and deep learning (DL) methods. **(A)** Type 1 model symmetry plane. **(B)** Type 2 model parent artery symmetry plane. **(C)** Type 2 model aneurysm cross section.

**FIGURE 6:**
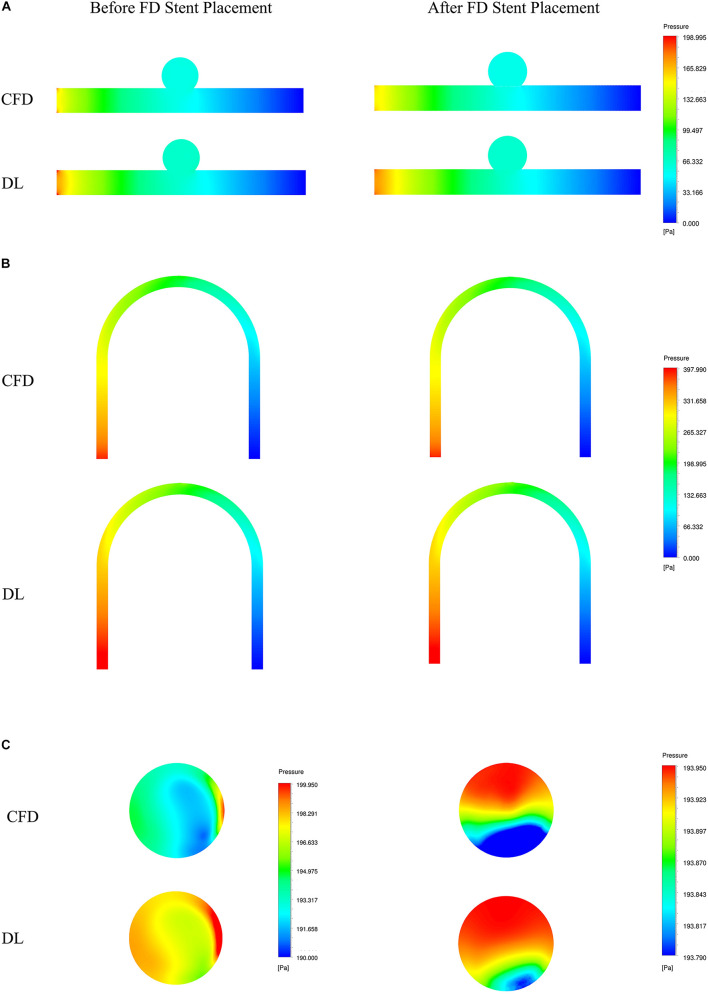
Comparison of pressure fields from CFD and deep learning (DL) methods. **(A)** Type 1 model symmetry plane. **(B)** Type 2 model parent artery symmetry plane. **(C)** Type 2 model aneurysm cross section.

The velocity field and pressure field in the selected section are shown in Figures 5, 6. The accuracy of CFD simulation results—that was, the quality of data sets—directly affected the deep learning prediction results. In this study, the CFD results were consistent with previous simulation studies or case reports (Valen-Sendstad and Steinman, 2014; Chung and Cebral, 2015; Updegrove et al., 2017). The blood flow in the parent artery of aneurysm had parabolic velocity profile in the straight part, and according to the inertia, higher velocity distributed along to the curved parent vessel. There was complex circulation flow along the aneurysm wall. The aneurysm flow came into aneurysm at the distal side of the neck. The porous media model had a significant shunt effect, which meant the blood flow and velocity into the aneurysm were greatly reduced compared to before FD placement. The pressure distribution and the range of pressure drop inside the aneurysm were consistent with previous CFD studies (Steinman et al., 2013; Jansen et al., 2014; Abdelsamie et al., 2016). This consistency ensured that the data set used for deep learning was also accurate and of high quality.

Deep learning predicted results and CFD results were in good agreement. In terms of flow field properties, our deep learning method could not only predict the laminar properties of the blood flow in the parent artery, but also predict the generation of complex vortexes inside the aneurysm. In terms of the stage before and after FD stent placement, deep learning could predict the distribution of flow velocity and pressure field before operation, and reflect its change after operation.

Table 3 shows the ERR statistics result of the internal flow velocity and pressure field of the test set models. In addition to the overall error of the model, we segmented the aneurysm part and calculated the corresponding error value separately. Our deep learning method had a good performance in hemodynamic prediction. All ERRs were smaller than 13%. The prediction error of velocity field was higher than that of the pressure field. This was because the velocity at each point has three components in x, y, and z directions. Compared with the pressure, the velocity computational burden of the network increased three times, which led to the increase of the ERRs. The ERRs of type 2 models were larger than that of type 1 models, which was due to the more complex flow of type 2 models (parent artery flow). The ERRs of aneurysms in type 1 and type 2 models were larger than that in the whole model. The value of ERRs was especially high in type 2 models’ aneurysms part. We provided a further explanation in the “Discussion” section.

**TABLE 3:** ERRs statistics results.

	**Model**	**Hemodynamics**	**Part**	**NMAE**	**MRE**
Preoperative	Type 1	Velocity	Whole model	3.46 ± 1.21	7.52 ± 1.33
			Aneurysm	3.71 ± 2.31	7.62 ± 2.01
		Pressure	Whole model	2.21 ± 1.35	6.35 ± 2.13
			Aneurysm	2.97 ± 2.31	7.01 ± 1.97
	Type 2	Velocity	Whole model	4.31 ± 1.95	9.81 ± 1.59
			Aneurysm	6.37 ± 2.81	12.97 ± 3.26
		Pressure	Whole model	3.54 ± 2.03	6.82 ± 2.51
			Aneurysm	5.01 ± 2.75	9.01 ± 3.12
Postoperative	Type 1	Velocity	Whole model	3.71 ± 2.01	7.84 ± 1.68
			Aneurysm	3.75 ± 1.38	8.01 ± 1.52
		Pressure	Whole model	2.17 ± 1.41	7.01 ± 1.69
			Aneurysm	2.21 ± 1.54	7.15 ± 1.34
	Type 2	Velocity	Whole model	4.27 ± 1.87	9.92 ± 1.98
			Aneurysm	5.01 ± 2.17	11.21 ± 2.10
		Pressure	Whole model	3.71 ± 2.21	6.94 ± 1.26
			Aneurysm	4.25 ± 1.98	7.31 ± 2.15

As for computational cost, hemodynamics of one model could be obtained within 1 s through deep learning. For the CFD method, including the construction and placement simulation operation of porous-medium model of the FD stent and calculation of hemodynamics, it took about 30 min on an Intel Xeon Gold 6148 2.4 GHz × 2 CPU server. Deep learning could reduce the calculation time 1,800 times.

## Discussion

In this study, we proposed a deep learning method to predict the hemodynamics of cerebral aneurysms before and after the FD stent placement. Compared with the previous deep learning method, our deep learning method could flexibly characterize the complex geometry including fluid domain and FD domain in the same scheme. And it realized the flow field prediction without identifying the point cloud belonging to FD domain. The results of error analysis showed that our deep learning method could achieve hemodynamic prediction with high accuracy (ERR < 13%) while reducing the calculation time by 1,800 times. It showed the practical value of deep learning in the task of calculating hemodynamics, which could greatly reduce the computational cost and simplify the operation process.

We conducted a comparative analysis of the previous studies on the prediction of flow field or hemodynamic parameters via machine or deep learning (Guo et al., 2016; Itu et al., 2016; Lee and You, 2019; Liang et al., 2020), as shown in Table 4.

**TABLE 4:** Flow or hemodynamics prediction accuracy reported by other machine or deep learning studies.

**Method**	**Output object**	**Data set size**	**Input data format**	**Error function or accuracy**
The proposed deep learning method	3D cerebral aneurysm hemodynamics	500	Flexible point cloud	NMAE < 6.5%, MRE < 13%
Itu’s machine-learning model	Fractional flow reserve (FFR) value	12,000	Geometric parameter	Error = 0.03%
Lee’s CNNs	2D unsteady flow field	500,000	Fixed meshes	32.8% < Error < 1%
Guo’s DCNNs	2D/3D steady flow	400,000	Fixed pixels	MRE < 3%
Liang’s DNNs	3D thoracic aorta hemodynamics	729	Fixed meshes	NMAE < 6.5%

The use of machine learning or deep learning for hemodynamic parameters (such as FFR) and flow field prediction was still limited. Itu et al.’s (2016) machine learning method could realize the prediction of FFR parameters. However, this kind of reduced order model had strong pertinence, and its application scope was limited. The convolution neural network model proposed by Guo et al. (2016) and Lee and You (2019) mainly aimed at the prediction of 2D flow field. The dimensionality reduction processing usually led to the loss of flow field information. For example, the velocity or pressure component in the direction perpendicular to the plane could not be predicted. Although Guo et al.’s study could realize simple 3D flow field prediction, its data normalization processing operation introduced a large amount of invalid information, which caused a large data set to exceed the calculation upper limit of the network. Take the cerebral aneurysm model used in this study as an example, if Guo et al.’s method was used, our model (Type 2) should be placed in a 3D rectangle with the length, width and height of 220 mm, 15 mm, and 200 mm (taking the maximum side length of all models) for normalization, respectively. One cerebral aneurysm model needed 2200 × 150 × 2000 = 660000000 pixels to represent the segmentation at 0.1 mm level, and more than half of them were normalized fill pixels with a parameter value of 0 (invalid zero padding information). Liang et al. (2020) used fixed meshes to realize model segmentation and normalization. They represented different 3D idealized thoracic aorta models as fixed meshes with only 80,100 nodes. However, previous studies showed that the number of meshes that could accurately characterize the spatial structure of the thoracic aorta should be dozens of times the result of Liang et al.’s (2020) division. Therefore, the resolution of the samples in its data set was limited. In this study, we used flexible point clouds to characterize the geometric information and internal flow field distribution of the cerebral aneurysm model. The point cloud could be directly extracted from the CFD calculation results without additional normalization processing. For one aneurysm model (type 2), the model point cloud included about 0.05 million points and the query point cloud included about 0.26 million points. The result of the mesh independent test proved that it was sufficient to characterize the spatial geometry of cerebral aneurysms with suitable resolution. The number and spatial coordinates of point clouds varied with different aneurysm models. This variability was conducive to accurately express the characteristics of different complex model, but it was also the data format that previous deep learning network cannot process. A double input and sampling channel deep learning network was proposed according to the properties of point cloud. The two channels could separately extract geometric information of model point cloud and hemodynamic information of query point cloud and stitched them together. With the global geometric information of the model as the constraint and the local hemodynamic information as the teacher’s signal, the network could effectively introduce the spatial relationship to realize the point-by-point hemodynamic prediction. Our deep learning method could characterize the spatial information and flow field distribution of the model in a flexible and accurate way. It could be extended to the hemodynamic prediction of models other than cerebral aneurysms, such as in our previous study: hemodynamics prediction before and after coronary artery bypass grafting. Considering the computational time, our deep learning method had a higher application value and scope, such as real-time guidance for different parts of surgery.

The ERRs of type 2 models were higher than that of type 1. This might be due to the different complexity of the parent artery flow field. To fully develop the flow field, we constructed a long parent artery. The number of parent artery query points accounted for about 90% of the whole model, which meant that the ERRs of the whole model were mainly determined by the parent artery. In type 2 models, secondary flow due to curvature happened in the parent artery (Sasaki et al., 2007; Anastasiou et al., 2012), whereas type 1 almost showed parabolic profile along the straight artery. This secondary flow in the parent artery of type 2 model resulted in higher complexity than the ideal laminar flow in the parent artery of type 1 model, which led to higher prediction difficulty of deep learning network and higher ERRs of type 2 models.

The ERRs of the aneurysm were higher than that of the whole model. The flow field inside the aneurysm was mainly determined by the inflow flux at the aneurysm orifice. As Imai et al. (2008) reported, the inflow flux at the aneurysm orifice were strongly influenced by the parent artery flow. For type 2 models, the inflow flux at the aneurysm orifice changes due to LA and CP parameters and the secondary flow in the parent artery both could cause great changes in the flow field inside the aneurysm (Anzai et al., 2014), which increased the difficulty of network prediction. On the other hand, compare with the parent artery, the flow velocity in the aneurysm was low and the range was small, which caused the ERRs at the aneurysm part to be more sensitive to changes in the flow field. Finally, the flow field pattern of the parent artery in type 1 and type 2 models was constant. Based on the above factors, the ERRs of the aneurysm were higher than the parent artery (the whole model), and the ERRs of the aneurysm part of type 2 models were the highest.

This study had several limitations. First, there was a lack of data for real patients’ cerebral aneurysms. We built the deep learning aneurysm data sets by combining morphological parameters to replace the real model. This inevitably led to the deviation of the optimal parameter configuration of the network obtained from the training samples from the real situation. Secondly, we only selected the side-wall aneurysms on cavernous branches as the object, and lack studies on other types of cerebral aneurysms (such as fusiform aneurysm, etc.). In further work, we will collect different types of cerebral aneurysm models from real patients to verify the applicability of our deep learning method. Thirdly, in the CFD simulation process, we used general boundary conditions instead of personalized boundary conditions. In addition, we used one kind of porous media model to replace the real stent. This may also lead to a potential risk, because the influence of the porous media model and the real stent model on hemodynamics was different. However, this parameter setting strategy was widely used in lots of simulation studies (Boutsianis et al., 2009; Martin, 2016; Vinoth et al., 2017). We focused on how to select a flexible data format to represent model geometry with high resolution and propose a corresponding network structure. In the following research, we will further propose deep learning methods under multiple constraints (such as patient specific unsteady flow boundary conditions, porous media model properties and geometries, etc.) to achieve more complex flow field predictions.

## Conclusion

In this study, we built flexible, high-resolution point cloud data sets and propose the corresponding network structure. On this basis, we realized the fast and accurate prediction of cerebral aneurysm hemodynamics before and after stent placement.

Compared with the traditional CFD method, our deep learning method can greatly simplify the operation process and reduce the computational cost. Compared with previous deep learning research, our deep learning method has more flexible data format and higher resolution, which means higher versatility and can be applied to the flow field prediction of other human parts or even other research fields. In terms of data resolution, computational efficiency, and universality, our deep learning method can meet the needs of most situations.

## Data Availability Statement

The raw data supporting the conclusions of this article will be made available by the authors, without undue reservation.

## Author Contributions

GL, XS, and XW created and designed this study. GL, HW, and SL performed the simulation and analyzed the data. All authors discussed and co-authored the manuscript.

## Conflict of Interest

The authors declare that the research was conducted in the absence of any commercial or financial relationships that could be construed as a potential conflict of interest.

## Publisher’s Note

All claims expressed in this article are solely those of the authors and do not necessarily represent those of their affiliated organizations, or those of the publisher, the editors and the reviewers. Any product that may be evaluated in this article, or claim that may be made by its manufacturer, is not guaranteed or endorsed by the publisher.
